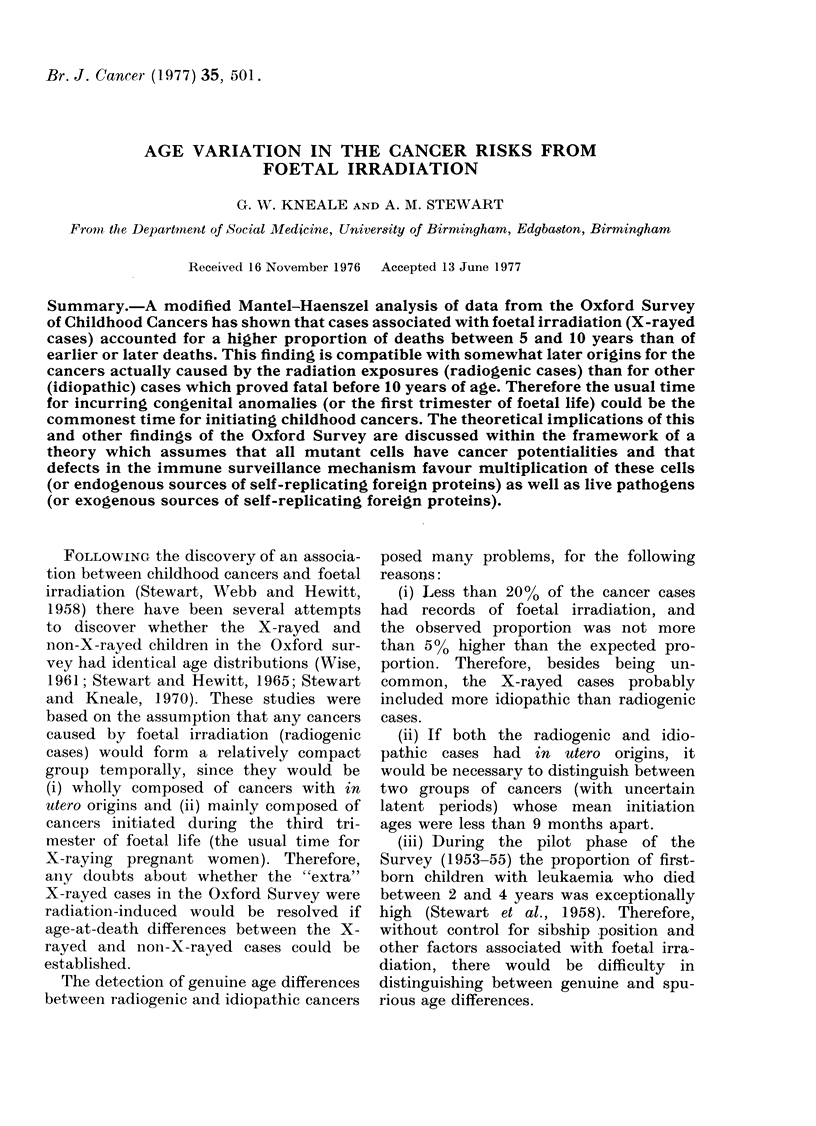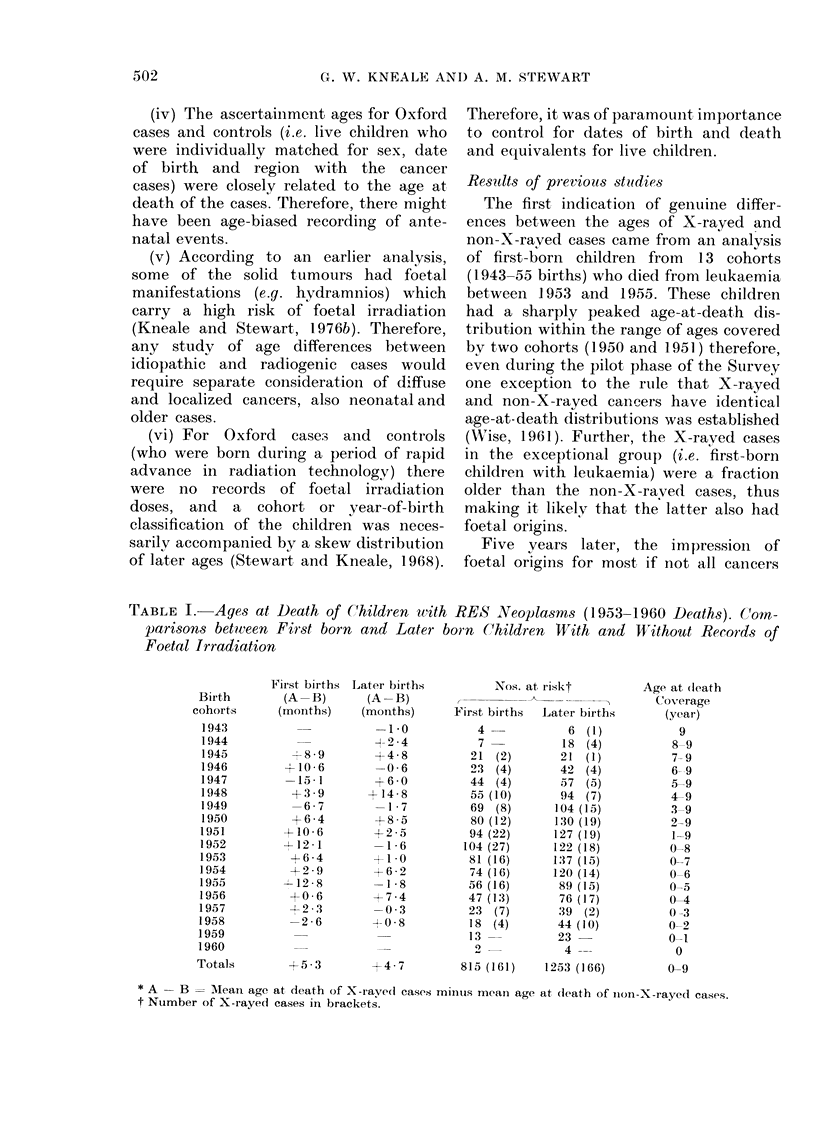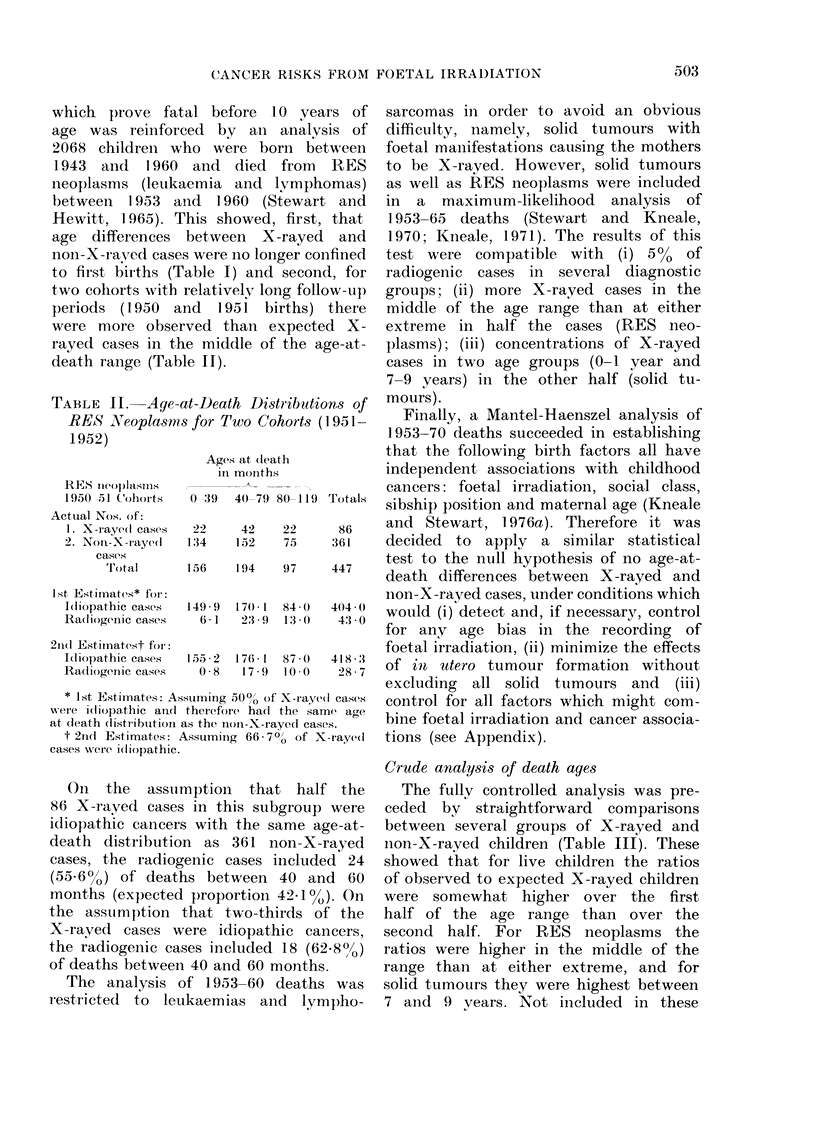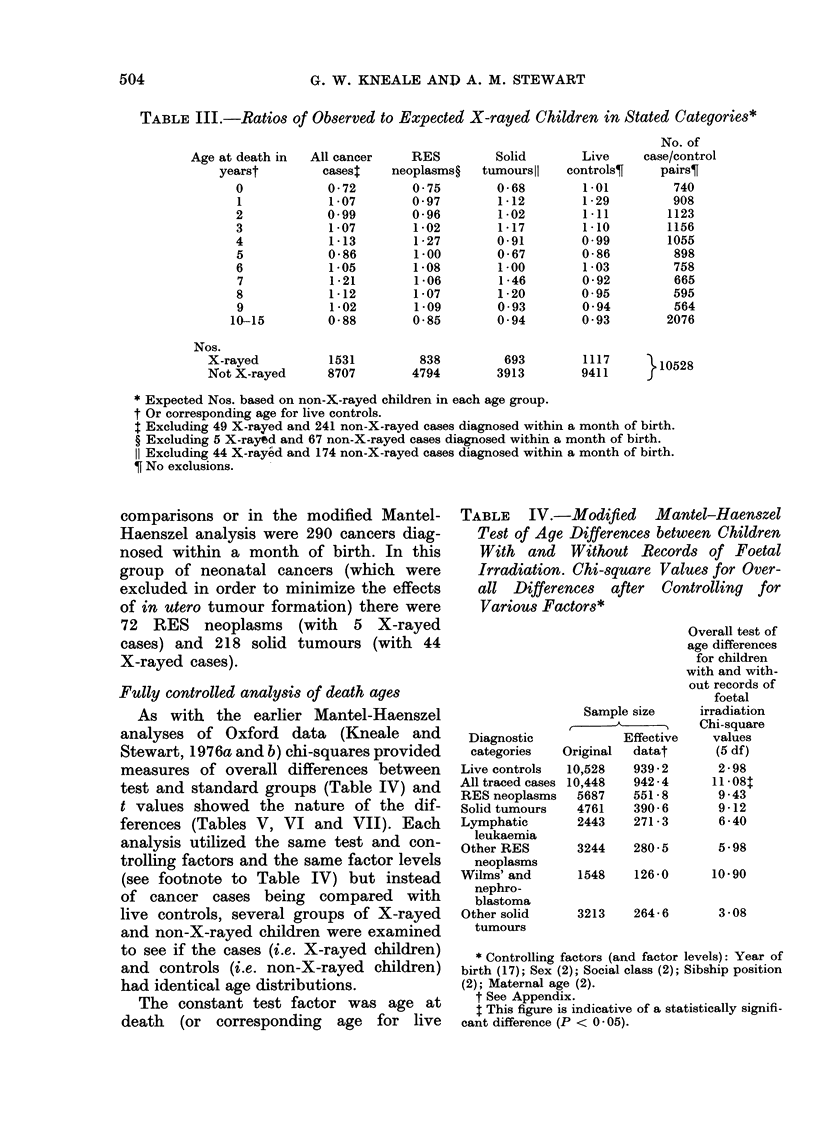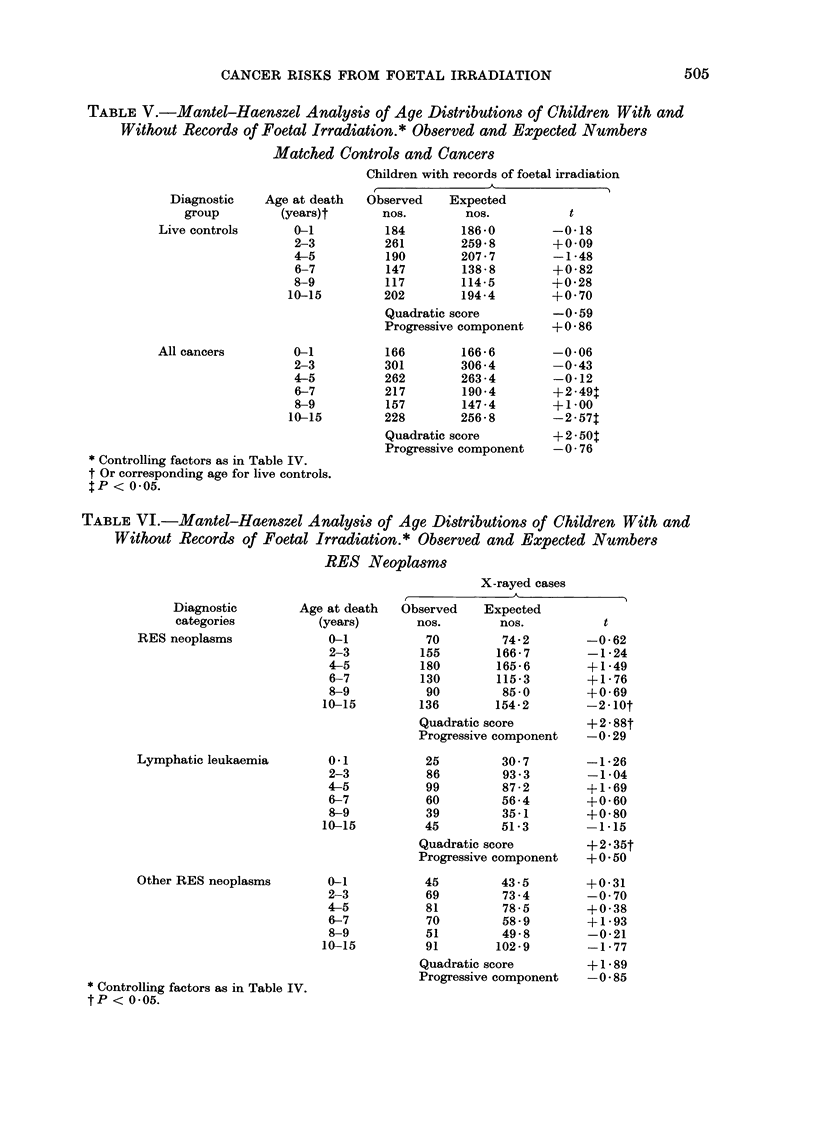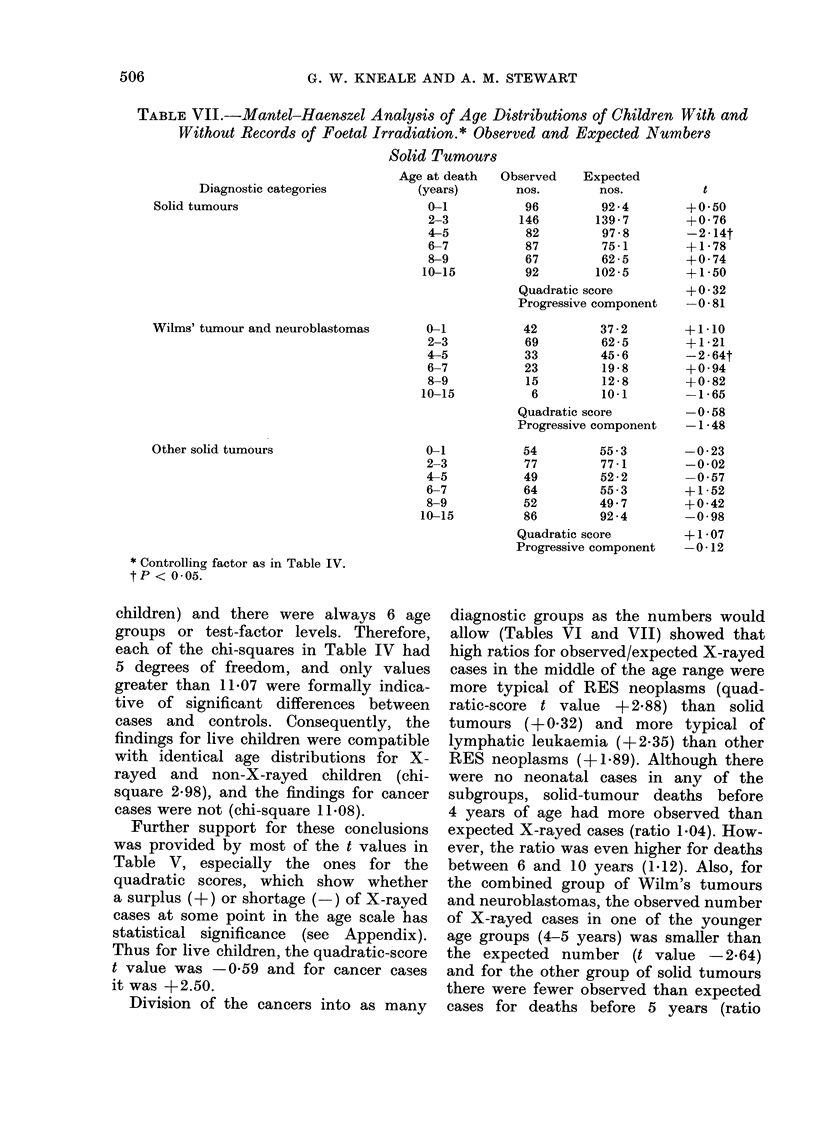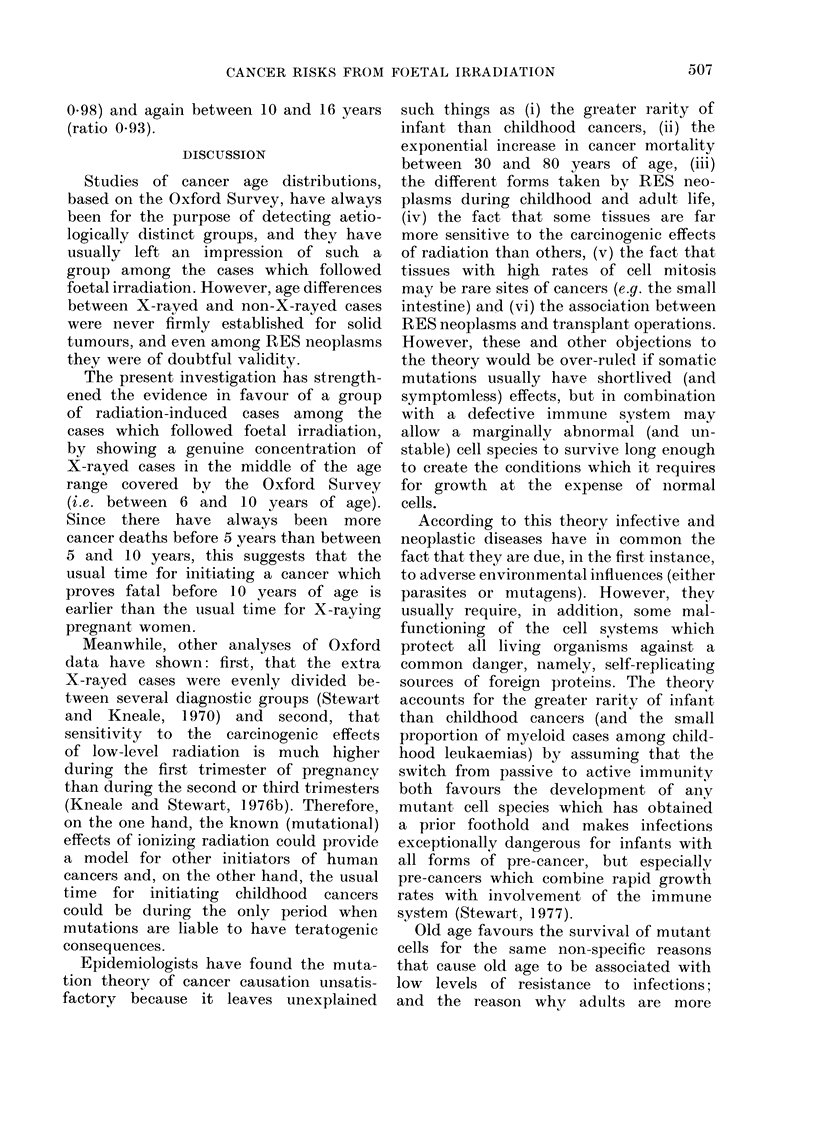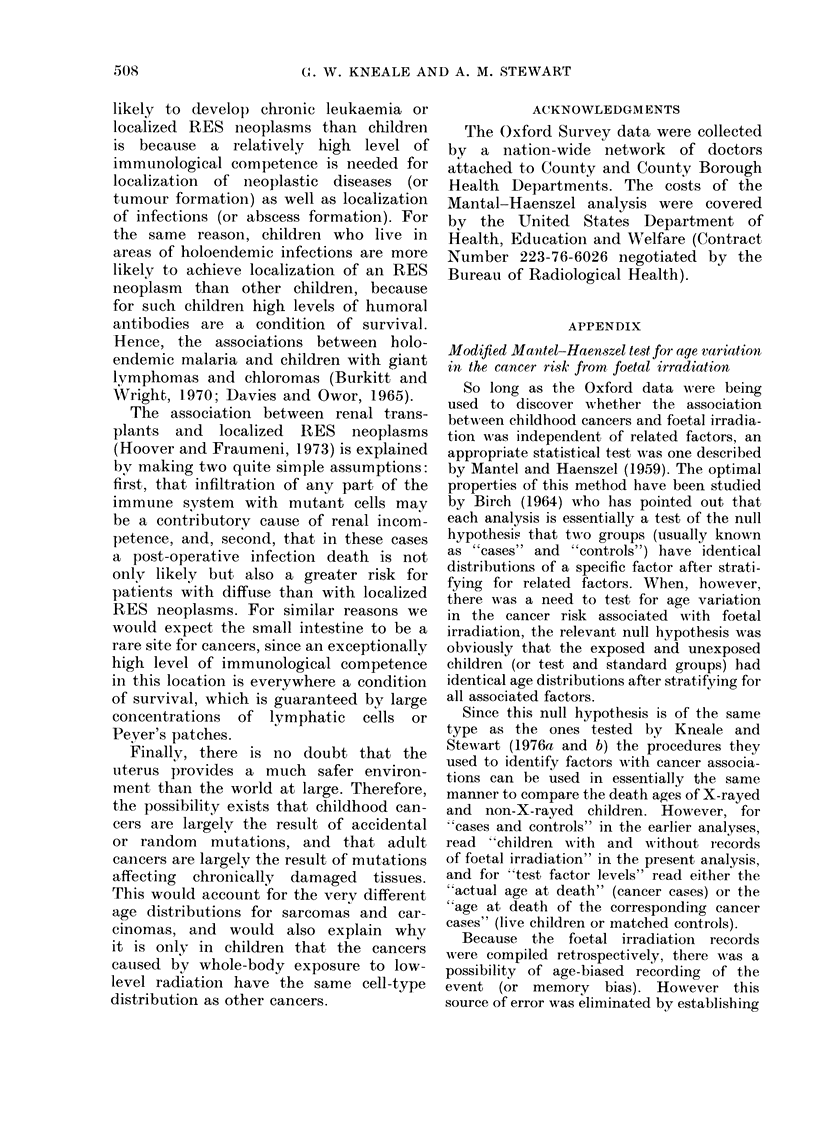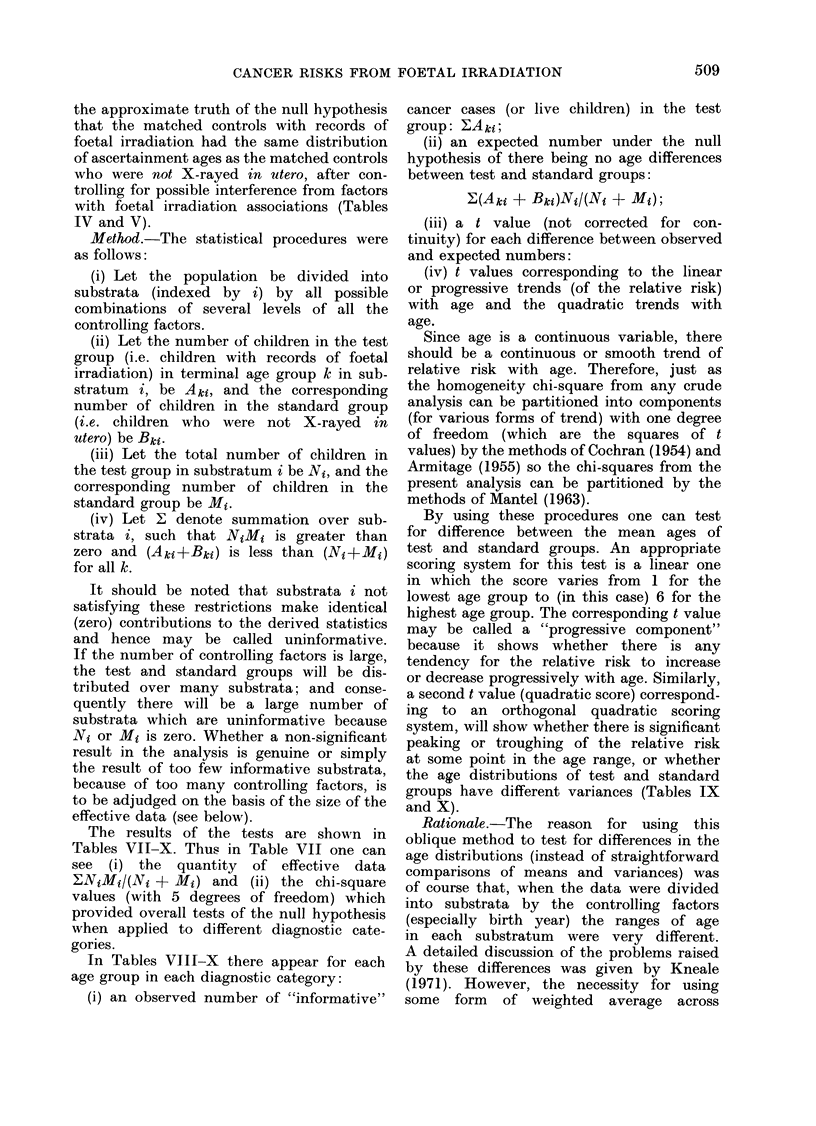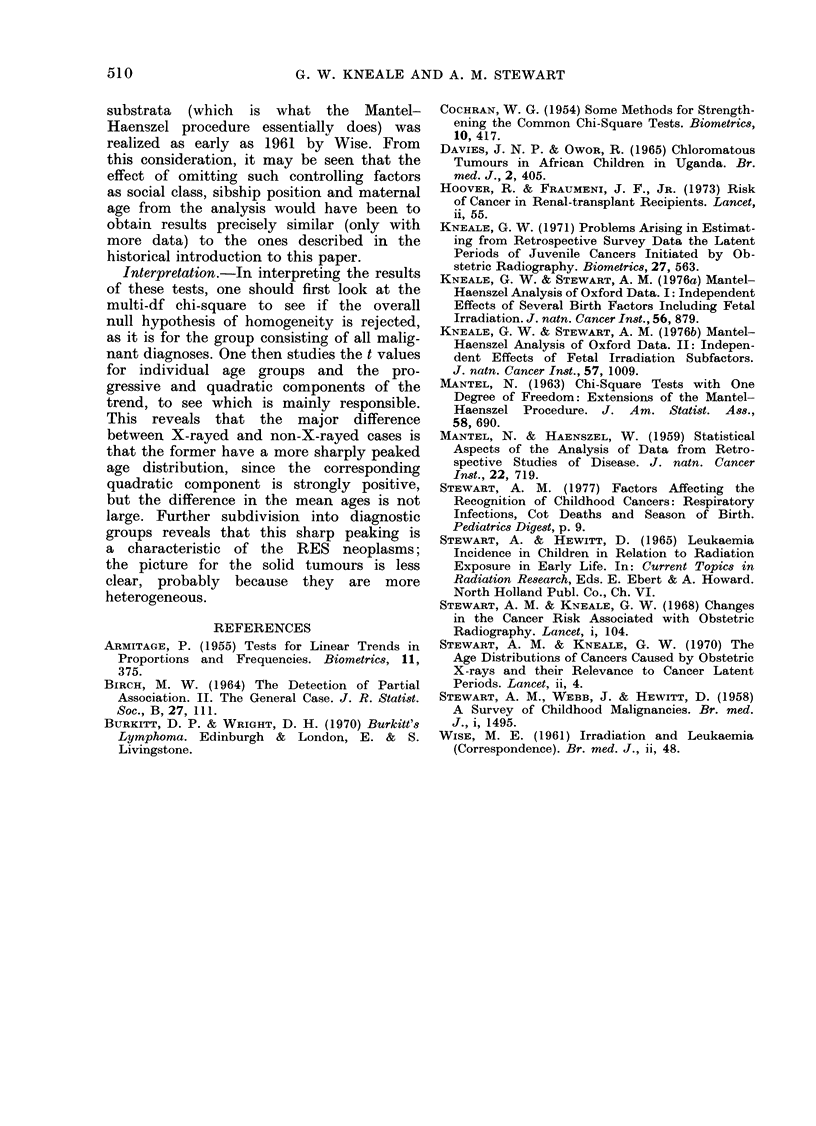# Age variation in the cancer risks from foetal irradiation.

**DOI:** 10.1038/bjc.1977.220

**Published:** 1977-10

**Authors:** G. W. Kneale, A. M. Stewart

## Abstract

A modified Mantel-Haenszel analysis of data from the Oxford Survey of Childhood Cancers has shown that cases associated with foetal irradiation (X-rayed cases) accounted for a higher proportion of deaths between 5 and 10 years than of earlier or later deaths. This finding is compatible with somewhat later origins for the cancers actually caused by the radiation exposures (radiogenic cases) than for other (idiopathic) cases which proved fatal before 10 years of age. Therefore the usual time for incurring congenital anomalies (or the first trimester of foetal life) could be the commonest time for initiating childhood cancers. The theoretical implications of this and other findings of the Oxford Survey are discussed within the framework of a theory which assumes that all mutant cells have cancer potentialities and that defects in the immune surveillance mechanism favour multiplication of these cells (or endogenous sources of self-replicating foreign proteins) as well as live pathogens (or exogenous sources of self-replicating foreign proteins).


*Br. J. Cancer* (1977) **35**, 501.

### AGE VARIATION IN THE CANCER RISKS FROM FOETAL IRRADIATION

G. W. KNEALE AND A. M. STEWART

*From the Department of Social Medicine, University of Birmingham, Edgbaston, Birmingham*

Received 16 November 1976  Accepted 13 June 1977

**Summary.—A modified Mantel–Haenszel analysis of data from the Oxford Survey of Childhood Cancers has shown that cases associated with foetal irradiation (X-rayed cases) accounted for a higher proportion of deaths between 5 and 10 years than of earlier or later deaths. This finding is compatible with somewhat later origins for the cancers actually caused by the radiation exposures (radiogenic cases) than for other (idiopathic) cases which proved fatal before 10 years of age. Therefore the usual time for incurring congenital anomalies (or the first trimester of foetal life) could be the commonest time for initiating childhood cancers. The theoretical implications of this and other findings of the Oxford Survey are discussed within the framework of a theory which assumes that all mutant cells have cancer potentialities and that defects in the immune surveillance mechanism favour multiplication of these cells (or endogenous sources of self-replicating foreign proteins) as well as live pathogens (or exogenous sources of self-replicating foreign proteins).**

FOLLOWING the discovery of an association between childhood cancers and foetal irradiation (Stewart, Webb and Hewitt, 1958) there have been several attempts to discover whether the X-rayed and non-X-rayed children in the Oxford survey had identical age distributions (Wise, 1961; Stewart and Hewitt, 1965; Stewart and Kneale, 1970). These studies were based on the assumption that any cancers caused by foetal irradiation (radiogenic cases) would form a relatively compact group temporally, since they would be (i) wholly composed of cancers with *in utero* origins and (ii) mainly composed of cancers initiated during the third trimester of foetal life (the usual time for X-raying pregnant women). Therefore, any doubts about whether the "extra" X-rayed cases in the Oxford Survey were radiation-induced would be resolved if age-at-death differences between the X-rayed and non-X-rayed cases could be established.

The detection of genuine age differences between radiogenic and idiopathic cancers posed many problems, for the following reasons:

(i) Less than 20% of the cancer cases had records of foetal irradiation, and the observed proportion was not more than 5% higher than the expected proportion. Therefore, besides being uncommon, the X-rayed cases probably included more idiopathic than radiogenic cases.

(ii) If both the radiogenic and idiopathic cases had *in utero* origins, it would be necessary to distinguish between two groups of cancers (with uncertain latent periods) whose mean initiation ages were less than 9 months apart.

(iii) During the pilot phase of the Survey (1953–55) the proportion of first-born children with leukaemia who died between 2 and 4 years was exceptionally high (Stewart *et al.*, 1958). Therefore, without control for sibship position and other factors associated with foetal irradiation, there would be difficulty in distinguishing between genuine and spurious age differences.

(iv) The ascertainment ages for Oxford cases and controls (*i.e.* live children who were individually matched for sex, date of birth and region with the cancer cases) were closely related to the age at death of the cases. Therefore, there might have been age-biased recording of antenatal events.

(v) According to an earlier analysis, some of the solid tumours had foetal manifestations (*e.g.* hydramnios) which carry a high risk of foetal irradiation (Kneale and Stewart, 1976*b*). Therefore, any study of age differences between idiopathic and radiogenic cases would require separate consideration of diffuse and localized cancers, also neonatal and older cases.

(vi) For Oxford cases and controls (who were born during a period of rapid advance in radiation technology) there were no records of foetal irradiation doses, and a cohort or year-of-birth classification of the children was necessarily accompanied by a skew distribution of later ages (Stewart and Kneale, 1968). Therefore, it was of paramount importance to control for dates of birth and death and equivalents for live children.

*Results of previous studies*

The first indication of genuine differences between the ages of X-rayed and non-X-rayed cases came from an analysis of first-born children from 13 cohorts (1943–55 births) who died from leukaemia between 1953 and 1955. These children had a sharply peaked age-at-death distribution within the range of ages covered by two cohorts (1950 and 1951) therefore, even during the pilot phase of the Survey one exception to the rule that X-rayed and non-X-rayed cancers have identical age-at-death distributions was established (Wise, 1961). Further, the X-rayed cases in the exceptional group (*i.e.* first-born children with leukaemia) were a fraction older than the non-X-rayed cases, thus making it likely that the latter also had foetal origins.

Five years later, the impression of foetal origins for most if not all cancers

TABLE I.—*Ages at Death of Children with RES Neoplasms* (1953–1960 *Deaths*). *Comparisons between First born and Later born Children With and Without Records of Foetal Irradiation*

| Birth cohorts | First births (A−B) (months) | Later births (A−B) (months) | Nos. at risk† First births | Nos. at risk† Later births | Age at death Coverage (year) |
|---|---|---|---|---|---|
| 1943 | — | −1·0 | 4 — | 6 (1) | 9 |
| 1944 | — | +2·4 | 7 — | 18 (4) | 8–9 |
| 1945 | +8·9 | +4·8 | 21 (2) | 21 (1) | 7–9 |
| 1946 | +10·6 | −0·6 | 23 (4) | 42 (4) | 6–9 |
| 1947 | −15·1 | +6·0 | 44 (4) | 57 (5) | 5–9 |
| 1948 | +3·9 | +14·8 | 55 (10) | 94 (7) | 4–9 |
| 1949 | −6·7 | −1·7 | 69 (8) | 104 (15) | 3–9 |
| 1950 | +6·4 | +8·5 | 80 (12) | 130 (19) | 2–9 |
| 1951 | +10·6 | +2·5 | 94 (22) | 127 (19) | 1–9 |
| 1952 | +12·1 | −1·6 | 104 (27) | 122 (18) | 0–8 |
| 1953 | +6·4 | +1·0 | 81 (16) | 137 (15) | 0–7 |
| 1954 | +2·9 | +6·2 | 74 (16) | 120 (14) | 0–6 |
| 1955 | +12·8 | −1·8 | 56 (16) | 89 (15) | 0–5 |
| 1956 | +0·6 | +7·4 | 47 (13) | 76 (17) | 0–4 |
| 1957 | +2·3 | −0·3 | 23 (7) | 39 (2) | 0–3 |
| 1958 | −2·6 | +0·8 | 18 (4) | 44 (10) | 0–2 |
| 1959 | — | — | 13 — | 23 — | 0–1 |
| 1960 | — | — | 2 — | 4 — | 0 |
| Totals | +5·3 | +4·7 | 815 (161) | 1253 (166) | 0–9 |

* A − B = Mean age at death of X-rayed cases minus mean age at death of non-X-rayed cases.

† Number of X-rayed cases in brackets.

which prove fatal before 10 years of age was reinforced by an analysis of 2068 children who were born between 1943 and 1960 and died from RES neoplasms (leukaemia and lymphomas) between 1953 and 1960 (Stewart and Hewitt, 1965). This showed, first, that age differences between X-rayed and non-X-rayed cases were no longer confined to first births (Table I) and second, for two cohorts with relatively long follow-up periods (1950 and 1951 births) there were more observed than expected X-rayed cases in the middle of the age-at-death range (Table II).

TABLE II.—*Age-at-Death Distributions of RES Neoplasms for Two Cohorts* (1951–1952)

| RES neoplasms 1950–51 Cohorts | Ages at death in months 0–39 | 40–79 | 80–119 | Totals |
|---|---|---|---|---|
| Actual Nos. of: | | | | |
| 1. X-rayed cases | 22 | 42 | 22 | 86 |
| 2. Non-X-rayed cases | 134 | 152 | 75 | 361 |
| Total | 156 | 194 | 97 | 447 |
| 1st Estimates* for: | | | | |
| Idiopathic cases | 149·9 | 170·1 | 84·0 | 404·0 |
| Radiogenic cases | 6·1 | 23·9 | 13·0 | 43·0 |
| 2nd Estimates† for: | | | | |
| Idiopathic cases | 155·2 | 176·1 | 87·0 | 418·3 |
| Radiogenic cases | 0·8 | 17·9 | 10·0 | 28·7 |

* 1st Estimates: Assuming 50% of X-rayed cases were idiopathic and therefore had the same age at death distribution as the non-X-rayed cases.

† 2nd Estimates: Assuming 66·7% of X-rayed cases were idiopathic.

On the assumption that half the 86 X-rayed cases in this subgroup were idiopathic cancers with the same age-at-death distribution as 361 non-X-rayed cases, the radiogenic cases included 24 (55·6%) of deaths between 40 and 60 months (expected proportion 42·1%). On the assumption that two-thirds of the X-rayed cases were idiopathic cancers, the radiogenic cases included 18 (62·8%) of deaths between 40 and 60 months.

The analysis of 1953–60 deaths was restricted to leukaemias and lymphosarcomas in order to avoid an obvious difficulty, namely, solid tumours with foetal manifestations causing the mothers to be X-rayed. However, solid tumours as well as RES neoplasms were included in a maximum-likelihood analysis of 1953–65 deaths (Stewart and Kneale, 1970; Kneale, 1971). The results of this test were compatible with (i) 5% of radiogenic cases in several diagnostic groups; (ii) more X-rayed cases in the middle of the age range than at either extreme in half the cases (RES neoplasms); (iii) concentrations of X-rayed cases in two age groups (0–1 year and 7–9 years) in the other half (solid tumours).

Finally, a Mantel-Haenszel analysis of 1953–70 deaths succeeded in establishing that the following birth factors all have independent associations with childhood cancers: foetal irradiation, social class, sibship position and maternal age (Kneale and Stewart, 1976*a*). Therefore it was decided to apply a similar statistical test to the null hypothesis of no age-at-death differences between X-rayed and non-X-rayed cases, under conditions which would (i) detect and, if necessary, control for any age bias in the recording of foetal irradiation, (ii) minimize the effects of *in utero* tumour formation without excluding all solid tumours and (iii) control for all factors which might combine foetal irradiation and cancer associations (see Appendix).

*Crude analysis of death ages*

The fully controlled analysis was preceded by straightforward comparisons between several groups of X-rayed and non-X-rayed children (Table III). These showed that for live children the ratios of observed to expected X-rayed children were somewhat higher over the first half of the age range than over the second half. For RES neoplasms the ratios were higher in the middle of the range than at either extreme, and for solid tumours they were highest between 7 and 9 years. Not included in these

TABLE III.—*Ratios of Observed to Expected X-rayed Children in Stated Categories**

| Age at death in years† | All cancer cases‡ | RES neoplasms§ | Solid tumours‖ | Live controls¶ | No. of case/control pairs¶ |
|---|---|---|---|---|---|
| 0 | 0·72 | 0·75 | 0·68 | 1·01 | 740 |
| 1 | 1·07 | 0·97 | 1·12 | 1·29 | 908 |
| 2 | 0·99 | 0·96 | 1·02 | 1·11 | 1123 |
| 3 | 1·07 | 1·02 | 1·17 | 1·10 | 1156 |
| 4 | 1·13 | 1·27 | 0·91 | 0·99 | 1055 |
| 5 | 0·86 | 1·00 | 0·67 | 0·86 | 898 |
| 6 | 1·05 | 1·08 | 1·00 | 1·03 | 758 |
| 7 | 1·21 | 1·06 | 1·46 | 0·92 | 665 |
| 8 | 1·12 | 1·07 | 1·20 | 0·95 | 595 |
| 9 | 1·02 | 1·09 | 0·93 | 0·94 | 564 |
| 10–15 | 0·88 | 0·85 | 0·94 | 0·93 | 2076 |
| Nos. | | | | | |
| X-rayed | 1531 | 838 | 693 | 1117 | } 10528 |
| Not X-rayed | 8707 | 4794 | 3913 | 9411 | |

* Expected Nos. based on non-X-rayed children in each age group.
† Or corresponding age for live controls.
‡ Excluding 49 X-rayed and 241 non-X-rayed cases diagnosed within a month of birth.
§ Excluding 5 X-rayed and 67 non-X-rayed cases diagnosed within a month of birth.
‖ Excluding 44 X-rayed and 174 non-X-rayed cases diagnosed within a month of birth.
¶ No exclusions.

comparisons or in the modified Mantel-Haenszel analysis were 290 cancers diagnosed within a month of birth. In this group of neonatal cancers (which were excluded in order to minimize the effects of *in utero* tumour formation) there were 72 RES neoplasms (with 5 X-rayed cases) and 218 solid tumours (with 44 X-rayed cases).

*Fully controlled analysis of death ages*

As with the earlier Mantel-Haenszel analyses of Oxford data (Kneale and Stewart, 1976*a* and *b*) chi-squares provided measures of overall differences between test and standard groups (Table IV) and *t* values showed the nature of the differences (Tables V, VI and VII). Each analysis utilized the same test and controlling factors and the same factor levels (see footnote to Table IV) but instead of cancer cases being compared with live controls, several groups of X-rayed and non-X-rayed children were examined to see if the cases (*i.e.* X-rayed children) and controls (*i.e.* non-X-rayed children) had identical age distributions.

The constant test factor was age at death (or corresponding age for live

TABLE IV.—*Modified Mantel–Haenszel Test of Age Differences between Children With and Without Records of Foetal Irradiation. Chi-square Values for Overall Differences after Controlling for Various Factors**

| Diagnostic categories | Sample size: Original | Sample size: Effective data† | Overall test of age differences for children with and without records of foetal irradiation Chi-square values (5 df) |
|---|---|---|---|
| Live controls | 10,528 | 939·2 | 2·98 |
| All traced cases | 10,448 | 942·4 | 11·08‡ |
| RES neoplasms | 5687 | 551·8 | 9·43 |
| Solid tumours | 4761 | 390·6 | 9·12 |
| Lymphatic leukaemia | 2443 | 271·3 | 6·40 |
| Other RES neoplasms | 3244 | 280·5 | 5·98 |
| Wilms' and nephroblastoma | 1548 | 126·0 | 10·90 |
| Other solid tumours | 3213 | 264·6 | 3·08 |

* Controlling factors (and factor levels): Year of birth (17); Sex (2); Social class (2); Sibship position (2); Maternal age (2).
† See Appendix.
‡ This figure is indicative of a statistically significant difference ($P < 0 \cdot 05$).

TABLE V.—*Mantel–Haenszel Analysis of Age Distributions of Children With and Without Records of Foetal Irradiation.* Observed and Expected Numbers*

*Matched Controls and Cancers*

| Diagnostic group | Age at death (years)† | Children with records of foetal irradiation: Observed nos. | Expected nos. | $t$ |
|---|---|---|---|---|
| Live controls | 0–1 | 184 | 186·0 | −0·18 |
| | 2–3 | 261 | 259·8 | +0·09 |
| | 4–5 | 190 | 207·7 | −1·48 |
| | 6–7 | 147 | 138·8 | +0·82 |
| | 8–9 | 117 | 114·5 | +0·28 |
| | 10–15 | 202 | 194·4 | +0·70 |
| | | Quadratic score | | −0·59 |
| | | Progressive component | | +0·86 |
| All cancers | 0–1 | 166 | 166·6 | −0·06 |
| | 2–3 | 301 | 306·4 | −0·43 |
| | 4–5 | 262 | 263·4 | −0·12 |
| | 6–7 | 217 | 190·4 | +2·49‡ |
| | 8–9 | 157 | 147·4 | +1·00 |
| | 10–15 | 228 | 256·8 | −2·57‡ |
| | | Quadratic score | | +2·50‡ |
| | | Progressive component | | −0·76 |

* Controlling factors as in Table IV.
† Or corresponding age for live controls.
‡ $P < 0 \cdot 05$.

TABLE VI.—*Mantel–Haenszel Analysis of Age Distributions of Children With and Without Records of Foetal Irradiation.* Observed and Expected Numbers*

*RES Neoplasms*

| Diagnostic categories | Age at death (years) | X-rayed cases: Observed nos. | Expected nos. | $t$ |
|---|---|---|---|---|
| RES neoplasms | 0–1 | 70 | 74·2 | −0·62 |
| | 2–3 | 155 | 166·7 | −1·24 |
| | 4–5 | 180 | 165·6 | +1·49 |
| | 6–7 | 130 | 115·3 | +1·76 |
| | 8–9 | 90 | 85·0 | +0·69 |
| | 10–15 | 136 | 154·2 | −2·10† |
| | | Quadratic score | | +2·88† |
| | | Progressive component | | −0·29 |
| Lymphatic leukaemia | 0·1 | 25 | 30·7 | −1·26 |
| | 2–3 | 86 | 93·3 | −1·04 |
| | 4–5 | 99 | 87·2 | +1·69 |
| | 6–7 | 60 | 56·4 | +0·60 |
| | 8–9 | 39 | 35·1 | +0·80 |
| | 10–15 | 45 | 51·3 | −1·15 |
| | | Quadratic score | | +2·35† |
| | | Progressive component | | +0·50 |
| Other RES neoplasms | 0–1 | 45 | 43·5 | +0·31 |
| | 2–3 | 69 | 73·4 | −0·70 |
| | 4–5 | 81 | 78·5 | +0·38 |
| | 6–7 | 70 | 58·9 | +1·93 |
| | 8–9 | 51 | 49·8 | −0·21 |
| | 10–15 | 91 | 102·9 | −1·77 |
| | | Quadratic score | | +1·89 |
| | | Progressive component | | −0·85 |

* Controlling factors as in Table IV.
† $P < 0 \cdot 05$.

TABLE VII.—*Mantel–Haenszel Analysis of Age Distributions of Children With and Without Records of Foetal Irradiation.* Observed and Expected Numbers*

*Solid Tumours*

| Diagnostic categories | Age at death (years) | Observed nos. | Expected nos. | t |
|---|---|---|---|---|
| Solid tumours | 0–1 | 96 | 92·4 | +0·50 |
| | 2–3 | 146 | 139·7 | +0·76 |
| | 4–5 | 82 | 97·8 | −2·14† |
| | 6–7 | 87 | 75·1 | +1·78 |
| | 8–9 | 67 | 62·5 | +0·74 |
| | 10–15 | 92 | 102·5 | +1·50 |
| | | Quadratic score | | +0·32 |
| | | Progressive component | | −0·81 |
| Wilms' tumour and neuroblastomas | 0–1 | 42 | 37·2 | +1·10 |
| | 2–3 | 69 | 62·5 | +1·21 |
| | 4–5 | 33 | 45·6 | −2·64† |
| | 6–7 | 23 | 19·8 | +0·94 |
| | 8–9 | 15 | 12·8 | +0·82 |
| | 10–15 | 6 | 10·1 | −1·65 |
| | | Quadratic score | | −0·58 |
| | | Progressive component | | −1·48 |
| Other solid tumours | 0–1 | 54 | 55·3 | −0·23 |
| | 2–3 | 77 | 77·1 | −0·02 |
| | 4–5 | 49 | 52·2 | −0·57 |
| | 6–7 | 64 | 55·3 | +1·52 |
| | 8–9 | 52 | 49·7 | +0·42 |
| | 10–15 | 86 | 92·4 | −0·98 |
| | | Quadratic score | | +1·07 |
| | | Progressive component | | −0·12 |

* Controlling factor as in Table IV.
† $P < 0{\cdot}05$.

children) and there were always 6 age groups or test-factor levels. Therefore, each of the chi-squares in Table IV had 5 degrees of freedom, and only values greater than 11·07 were formally indicative of significant differences between cases and controls. Consequently, the findings for live children were compatible with identical age distributions for X-rayed and non-X-rayed children (chi-square 2·98), and the findings for cancer cases were not (chi-square 11·08).

Further support for these conclusions was provided by most of the $t$ values in Table V, especially the ones for the quadratic scores, which show whether a surplus (+) or shortage (−) of X-rayed cases at some point in the age scale has statistical significance (see Appendix). Thus for live children, the quadratic-score $t$ value was −0·59 and for cancer cases it was +2.50.

Division of the cancers into as many diagnostic groups as the numbers would allow (Tables VI and VII) showed that high ratios for observed/expected X-rayed cases in the middle of the age range were more typical of RES neoplasms (quadratic-score $t$ value +2·88) than solid tumours (+0·32) and more typical of lymphatic leukaemia (+2·35) than other RES neoplasms (+1·89). Although there were no neonatal cases in any of the subgroups, solid-tumour deaths before 4 years of age had more observed than expected X-rayed cases (ratio 1·04). However, the ratio was even higher for deaths between 6 and 10 years (1·12). Also, for the combined group of Wilm's tumours and neuroblastomas, the observed number of X-rayed cases in one of the younger age groups (4–5 years) was smaller than the expected number ($t$ value −2·64) and for the other group of solid tumours there were fewer observed than expected cases for deaths before 5 years (ratio

0·98) and again between 10 and 16 years (ratio 0·93).

### DISCUSSION

Studies of cancer age distributions, based on the Oxford Survey, have always been for the purpose of detecting aetiologically distinct groups, and they have usually left an impression of such a group among the cases which followed foetal irradiation. However, age differences between X-rayed and non-X-rayed cases were never firmly established for solid tumours, and even among RES neoplasms they were of doubtful validity.

The present investigation has strengthened the evidence in favour of a group of radiation-induced cases among the cases which followed foetal irradiation, by showing a genuine concentration of X-rayed cases in the middle of the age range covered by the Oxford Survey (*i.e.* between 6 and 10 years of age). Since there have always been more cancer deaths before 5 years than between 5 and 10 years, this suggests that the usual time for initiating a cancer which proves fatal before 10 years of age is earlier than the usual time for X-raying pregnant women.

Meanwhile, other analyses of Oxford data have shown: first, that the extra X-rayed cases were evenly divided between several diagnostic groups (Stewart and Kneale, 1970) and second, that sensitivity to the carcinogenic effects of low-level radiation is much higher during the first trimester of pregnancy than during the second or third trimesters (Kneale and Stewart, 1976b). Therefore, on the one hand, the known (mutational) effects of ionizing radiation could provide a model for other initiators of human cancers and, on the other hand, the usual time for initiating childhood cancers could be during the only period when mutations are liable to have teratogenic consequences.

Epidemiologists have found the mutation theory of cancer causation unsatisfactory because it leaves unexplained such things as (i) the greater rarity of infant than childhood cancers, (ii) the exponential increase in cancer mortality between 30 and 80 years of age, (iii) the different forms taken by RES neoplasms during childhood and adult life, (iv) the fact that some tissues are far more sensitive to the carcinogenic effects of radiation than others, (v) the fact that tissues with high rates of cell mitosis may be rare sites of cancers (*e.g.* the small intestine) and (vi) the association between RES neoplasms and transplant operations. However, these and other objections to the theory would be over-ruled if somatic mutations usually have shortlived (and symptomless) effects, but in combination with a defective immune system may allow a marginally abnormal (and unstable) cell species to survive long enough to create the conditions which it requires for growth at the expense of normal cells.

According to this theory infective and neoplastic diseases have in common the fact that they are due, in the first instance, to adverse environmental influences (either parasites or mutagens). However, they usually require, in addition, some malfunctioning of the cell systems which protect all living organisms against a common danger, namely, self-replicating sources of foreign proteins. The theory accounts for the greater rarity of infant than childhood cancers (and the small proportion of myeloid cases among childhood leukaemias) by assuming that the switch from passive to active immunity both favours the development of any mutant cell species which has obtained a prior foothold and makes infections exceptionally dangerous for infants with all forms of pre-cancer, but especially pre-cancers which combine rapid growth rates with involvement of the immune system (Stewart, 1977).

Old age favours the survival of mutant cells for the same non-specific reasons that cause old age to be associated with low levels of resistance to infections; and the reason why adults are more likely to develop chronic leukaemia or localized RES neoplasms than children is because a relatively high level of immunological competence is needed for localization of neoplastic diseases (or tumour formation) as well as localization of infections (or abscess formation). For the same reason, children who live in areas of holoendemic infections are more likely to achieve localization of an RES neoplasm than other children, because for such children high levels of humoral antibodies are a condition of survival. Hence, the associations between holoendemic malaria and children with giant lymphomas and chloromas (Burkitt and Wright, 1970; Davies and Owor, 1965).

The association between renal transplants and localized RES neoplasms (Hoover and Fraumeni, 1973) is explained by making two quite simple assumptions: first, that infiltration of any part of the immune system with mutant cells may be a contributory cause of renal incompetence, and, second, that in these cases a post-operative infection death is not only likely but also a greater risk for patients with diffuse than with localized RES neoplasms. For similar reasons we would expect the small intestine to be a rare site for cancers, since an exceptionally high level of immunological competence in this location is everywhere a condition of survival, which is guaranteed by large concentrations of lymphatic cells or Peyer's patches.

Finally, there is no doubt that the uterus provides a much safer environment than the world at large. Therefore, the possibility exists that childhood cancers are largely the result of accidental or random mutations, and that adult cancers are largely the result of mutations affecting chronically damaged tissues. This would account for the very different age distributions for sarcomas and carcinomas, and would also explain why it is only in children that the cancers caused by whole-body exposure to low-level radiation have the same cell-type distribution as other cancers.

ACKNOWLEDGMENTS

The Oxford Survey data were collected by a nation-wide network of doctors attached to County and County Borough Health Departments. The costs of the Mantal–Haenszel analysis were covered by the United States Department of Health, Education and Welfare (Contract Number 223-76-6026 negotiated by the Bureau of Radiological Health).

### APPENDIX

*Modified Mantel–Haenszel test for age variation in the cancer risk from foetal irradiation*

So long as the Oxford data were being used to discover whether the association between childhood cancers and foetal irradiation was independent of related factors, an appropriate statistical test was one described by Mantel and Haenszel (1959). The optimal properties of this method have been studied by Birch (1964) who has pointed out that each analysis is essentially a test of the null hypothesis that two groups (usually known as "cases" and "controls") have identical distributions of a specific factor after stratifying for related factors. When, however, there was a need to test for age variation in the cancer risk associated with foetal irradiation, the relevant null hypothesis was obviously that the exposed and unexposed children (or test and standard groups) had identical age distributions after stratifying for all associated factors.

Since this null hypothesis is of the same type as the ones tested by Kneale and Stewart (1976*a* and *b*) the procedures they used to identify factors with cancer associations can be used in essentially the same manner to compare the death ages of X-rayed and non-X-rayed children. However, for "cases and controls" in the earlier analyses, read "children with and without records of foetal irradiation" in the present analysis, and for "test factor levels" read either the "actual age at death" (cancer cases) or the "age at death of the corresponding cancer cases" (live children or matched controls).

Because the foetal irradiation records were compiled retrospectively, there was a possibility of age-biased recording of the event (or memory bias). However this source of error was eliminated by establishing the approximate truth of the null hypothesis that the matched controls with records of foetal irradiation had the same distribution of ascertainment ages as the matched controls who were *not* X-rayed *in utero*, after controlling for possible interference from factors with foetal irradiation associations (Tables IV and V).

*Method*.—The statistical procedures were as follows:

(i) Let the population be divided into substrata (indexed by $i$) by all possible combinations of several levels of all the controlling factors.

(ii) Let the number of children in the test group (i.e. children with records of foetal irradiation) in terminal age group $k$ in substratum $i$, be $A_{ki}$, and the corresponding number of children in the standard group (*i.e.* children who were not X-rayed *in utero*) be $B_{ki}$.

(iii) Let the total number of children in the test group in substratum $i$ be $N_i$, and the corresponding number of children in the standard group be $M_i$.

(iv) Let $\Sigma$ denote summation over substrata $i$, such that $N_iM_i$ is greater than zero and $(A_{ki}+B_{ki})$ is less than $(N_i+M_i)$ for all $k$.

It should be noted that substrata $i$ not satisfying these restrictions make identical (zero) contributions to the derived statistics and hence may be called uninformative. If the number of controlling factors is large, the test and standard groups will be distributed over many substrata; and consequently there will be a large number of substrata which are uninformative because $N_i$ or $M_i$ is zero. Whether a non-significant result in the analysis is genuine or simply the result of too few informative substrata, because of too many controlling factors, is to be adjudged on the basis of the size of the effective data (see below).

The results of the tests are shown in Tables VII–X. Thus in Table VII one can see (i) the quantity of effective data $\Sigma N_iM_i/(N_i + M_i)$ and (ii) the chi-square values (with 5 degrees of freedom) which provided overall tests of the null hypothesis when applied to different diagnostic categories.

In Tables VIII–X there appear for each age group in each diagnostic category:

(i) an observed number of "informative" cancer cases (or live children) in the test group: $\Sigma A_{ki}$;

(ii) an expected number under the null hypothesis of there being no age differences between test and standard groups:

$$\Sigma(A_{ki} + B_{ki})N_i/(N_i + M_i);$$

(iii) a $t$ value (not corrected for continuity) for each difference between observed and expected numbers:

(iv) $t$ values corresponding to the linear or progressive trends (of the relative risk) with age and the quadratic trends with age.

Since age is a continuous variable, there should be a continuous or smooth trend of relative risk with age. Therefore, just as the homogeneity chi-square from any crude analysis can be partitioned into components (for various forms of trend) with one degree of freedom (which are the squares of $t$ values) by the methods of Cochran (1954) and Armitage (1955) so the chi-squares from the present analysis can be partitioned by the methods of Mantel (1963).

By using these procedures one can test for difference between the mean ages of test and standard groups. An appropriate scoring system for this test is a linear one in which the score varies from 1 for the lowest age group to (in this case) 6 for the highest age group. The corresponding $t$ value may be called a "progressive component" because it shows whether there is any tendency for the relative risk to increase or decrease progressively with age. Similarly, a second $t$ value (quadratic score) corresponding to an orthogonal quadratic scoring system, will show whether there is significant peaking or troughing of the relative risk at some point in the age range, or whether the age distributions of test and standard groups have different variances (Tables IX and X).

*Rationale*.—The reason for using this oblique method to test for differences in the age distributions (instead of straightforward comparisons of means and variances) was of course that, when the data were divided into substrata by the controlling factors (especially birth year) the ranges of age in each substratum were very different. A detailed discussion of the problems raised by these differences was given by Kneale (1971). However, the necessity for using some form of weighted average across substrata (which is what the Mantel–Haenszel procedure essentially does) was realized as early as 1961 by Wise. From this consideration, it may be seen that the effect of omitting such controlling factors as social class, sibship position and maternal age from the analysis would have been to obtain results precisely similar (only with more data) to the ones described in the historical introduction to this paper.

*Interpretation.*—In interpreting the results of these tests, one should first look at the multi-df chi-square to see if the overall null hypothesis of homogeneity is rejected, as it is for the group consisting of all malignant diagnoses. One then studies the *t* values for individual age groups and the progressive and quadratic components of the trend, to see which is mainly responsible. This reveals that the major difference between X-rayed and non-X-rayed cases is that the former have a more sharply peaked age distribution, since the corresponding quadratic component is strongly positive, but the difference in the mean ages is not large. Further subdivision into diagnostic groups reveals that this sharp peaking is a characteristic of the RES neoplasms; the picture for the solid tumours is less clear, probably because they are more heterogeneous.

### REFERENCES

Armitage, P. (1955) Tests for Linear Trends in Proportions and Frequencies. *Biometrics*, **11**, 375.

Birch, M. W. (1964) The Detection of Partial Association. II. The General Case. *J. R. Statist. Soc.*, B, **27**, 111.

Burkitt, D. P. & Wright, D. H. (1970) *Burkitt's Lymphoma.* Edinburgh & London, E. & S. Livingstone.

Cochran, W. G. (1954) Some Methods for Strengthening the Common Chi-Square Tests. *Biometrics*, **10**, 417.

Davies, J. N. P. & Owor, R. (1965) Chloromatous Tumours in African Children in Uganda. *Br. med. J.*, **2**, 405.

Hoover, R. & Fraumeni, J. F., Jr. (1973) Risk of Cancer in Renal-transplant Recipients. *Lancet*, ii, 55.

Kneale, G. W. (1971) Problems Arising in Estimating from Retrospective Survey Data the Latent Periods of Juvenile Cancers Initiated by Obstetric Radiography. *Biometrics*, **27**, 563.

Kneale, G. W. & Stewart, A. M. (1976*a*) Mantel–Haenszel Analysis of Oxford Data. I: Independent Effects of Several Birth Factors Including Fetal Irradiation. *J. natn. Cancer Inst.*, **56**, 879.

Kneale, G. W. & Stewart, A. M. (1976*b*) Mantel–Haenszel Analysis of Oxford Data. II: Independent Effects of Fetal Irradiation Subfactors. *J. natn. Cancer Inst.*, **57**, 1009.

Mantel, N. (1963) Chi-Square Tests with One Degree of Freedom: Extensions of the Mantel–Haenszel Procedure. *J. Am. Statist. Ass.*, **58**, 690.

Mantel, N. & Haenszel, W. (1959) Statistical Aspects of the Analysis of Data from Retrospective Studies of Disease. *J. natn. Cancer Inst.*, **22**, 719.

Stewart, A. M. (1977) Factors Affecting the Recognition of Childhood Cancers: Respiratory Infections, Cot Deaths and Season of Birth. *Pediatrics Digest*, p. 9.

Stewart, A. & Hewitt, D. (1965) Leukaemia Incidence in Children in Relation to Radiation Exposure in Early Life. In: *Current Topics in Radiation Research*, Eds. E. Ebert & A. Howard. North Holland Publ. Co., Ch. VI.

Stewart, A. M. & Kneale, G. W. (1968) Changes in the Cancer Risk Associated with Obstetric Radiography. *Lancet*, i, 104.

Stewart, A. M. & Kneale, G. W. (1970) The Age Distributions of Cancers Caused by Obstetric X-rays and their Relevance to Cancer Latent Periods. *Lancet*, ii, 4.

Stewart, A. M., Webb, J. & Hewitt, D. (1958) A Survey of Childhood Malignancies. *Br. med. J.*, i, 1495.

Wise, M. E. (1961) Irradiation and Leukaemia (Correspondence). *Br. med. J.*, ii, 48.